# Towards a spatially resolved, single-ended TDLAS system for characterizing the distribution of gaseous species

**DOI:** 10.1038/s41598-024-61644-9

**Published:** 2024-05-22

**Authors:** C. Hansemann, M. Bonarens, J. Emmert, K. J. Daun, S. Wagner

**Affiliations:** 1 Department of Mechanical Engineering, Reactive Flows and Diagnostics, Technical University of Darmstadt, Otto-Berndt-Str. 3, 64287 Darmstadt, Germany; 2 Department of Mechanical and Mechatronics Engineering, University of Waterloo, 200 University Avenue West, Waterloo, ON N2L 3G1 Canada

**Keywords:** Chemical engineering, Imaging and sensing, Optical techniques, Optical spectroscopy, Near-infrared spectroscopy

## Abstract

Many applications require diagnostics that can quantify the distribution of chemical gas species and gas temperature along a single line-of-sight, which is challenging in process environments with limited optical access. To this end, we present an approach that combines time-of-flight Light Detection and Ranging (LiDAR) with Tunable Diode Laser Absorption Spectroscopy (TDLAS) to scan individual gas molecular transition lines. This method is applicable in situations where scattering objects are distributed along the beam path, such as solid fuel combustion, or when dealing with multiple gas volumes separated by weakly reflecting windows. The approach is demonstrated through simulation studies and an initial experimental proof of concept for separated gas volumes.

## Introduction

Developing the next generation of low-emission, high-efficiency combustion devices rely on precise information on the distribution of gas species and gas temperature within the process. This, in turn, requires measurement methods that offer high spatial resolution while requiring minimal optical access. Established measurement techniques such as tomographic absorption spectroscopy^1,2^ and planar laser-induced fluorescence^3^ are well-suited for laboratory studies, but require a degree of optical access that is often unavailable for real-world devices. In certain cases, measurement systems exploiting the Scheimpflug principle for spatially resolved fluorescence or Rayleigh thermometry can be implemented to allow for more flexible design of the optical access^4^. In contrast, Tuneable Diode Laser Absorption Spectroscopy (TDLAS) demands significantly less optical access but provides only path-integrated information, which restricts comprehensive analysis along the line-of-sight (LOS)^5–8^.

To address this gap, absorption-based measurement approaches that exploit reflections or scattering within the probe volume, either by gas molecules, droplets, or solid-phase particles, to achieve spatial resolution have gained attention. These methods utilize the fact that light waves scattered at different points within the device traverse different paths in the investigated volume, and thereby contain information about the gas composition between the emitter and the scattering location. To infer local gas properties, it is necessary to disentangle the signal components based on where each wave was scattered. These scattering elements may constitute particles already present in the process, such as solid fuel, or additionally “seeded” particles or droplets, as common in particle image velocimetry. Scheimpflug LiDAR, which requires two optical access points and distinguishes signal components by their spatial offset, can only be applied if the displacement between the emitter and the detector is negligible compared to the detection range. This is because the traveled path of the scattered light is not identical with the path of the emitted radiation, which leads to entangled information that complicates the evaluation of absorbance signals if the gas state deviates between both paths. While the displacement may be negligible for long-range atmospheric measurements featuring smooth gradients in gas properties^9,10^, it is generally not negligible within combustion devices, which involves shorter distances and steep gradients in gas temperatures and composition.

One way to achieve single-ended, spatially resolved absorption spectroscopy is to merge absorption spectroscopy with time-of-flight ranging. In this approach time-modulated light pulse is emitted along the line of sight (LOS) and the resulting backscattered signal is recorded at high temporal resolution. The location at which a signal component is backscattered can be deduced from the time delay between emission and detection, while the attenuation of the signal component contains information on the properties of the flow along the LOS. The information about the gas and, if present, particle phase can be disentangled if measurements are performed with pulses of different wavelengths, since particles have a broadband influence on the back-reflected signal, while gases exhibit narrow absorption features. The local absorption properties of the gas can then be reconstructed spatially by inverting an integral equation. In this regard the spatial resolution depends on the distribution of reflectors along the LOS, the edge steepness of the laser pulse, and the sampling frequency of the data acquisition system.

Several existing diagnostics also use continuous-wave or pulsed lasers to infer the concentration distribution of a species along an optical path. Most differential absorption LiDAR (DIAL) systems use two laser pulses: one aligned to an absorption feature of the molecule of interest (“on resonant”) and one that is off-resonant. The backscattered signal from the on-resonant pulse is influenced by absorption and scattering, while the off-resonant backscattered pulse depends only on scattering. The distribution of molecular concentration along the optical path may then be inferred from the time-resolved ratio of the two back-scattered signals. A variation of this approach called Micro Pulse Lidar^11,12^ uses diode lasers to seed the amplifier, enabling long-term stable operation with precise narrowband spectral emission which is useful for filtering out background radiation. DIAL is particularly well suited for atmospheric research in different configurations^9,11–13^, and some proof-of-concept studies investigating lab-scale combustion have also been presented^14–16^. Conceptually, however, two-wavelength DIAL is prone to cross-sensitivities to other process parameters and requires calibration which may limit its applicability to combustors. Multi-frequency DIAL has recently gained increased interest due to its ability to perform measurements at different optical frequencies using tunable external cavity^17,18^ or current tuned diode laser^10^. These studies showcase the potential of multi-frequency DIAL in atmospheric research. Yu et al.^17^ demonstrated a system that chops a cavity length-tuned continuous wave (cw)-laser with an acousto-optical modulator to observe atmospheric CO_2_ spectra with a range resolution of 120 m. Stroud et al.^18^ successfully resolved 10 optical frequencies to probe CO_2_ and H_2_O. With a range resolution of 250 m, they can distinguish the combustion plume from a power plant from atmospheric background. Mei and Brydegaard^10^ used a tuneable diode laser in continuous wave (cw) operation in combination with an optical arrangement fulfilling the Scheimpflug condition to probe oxygen at seven frequencies with a range resolution in the order of 100 m. Hyperspectral LiDAR using a supercontinuum source has been demonstrated for material classification with high spatial resolution^19^. Bourdreau et al.^20^ used hyperspectral LiDAR based on frequency combs to analyze gas-phase absorption. While this is a promising approach, it is limited by discrete wavelength selection and optical bandwidth compared to supercontinuum sources, as well as spatial resolution^20^. Higher spectral resolution to scan specific transitions would be more favorable to simultaneously recover species concentration, temperature, and pressure.

In this article, we propose a novel measurement approach that combines TDLAS with optical ranging to achieve spatially resolved, single-ended detection of the gas phase properties for particle-laden gas configurations. Instead of hyperspectral sources, inexpensive tunable diode lasers are used as a slowly tunable source. The necessary steep intensity gradients for ToF ranging are introduced by an electro-optical modulator (EOM). Gas phase molecular absorption features are scanned by sequentially and repeatedly recording time resolved pulse reflection signals at different wavelengths. Differential, time-averaged gas phase absorbance spectra along the beam path can then be inferred following the DIAL principle. The fast rise time of the pulse combined with a high frequency data acquisition enables a spatial resolution in the order of 1 cm. First, we establish the theoretical foundations of this technique and then explore its potential by means of numerical simulations. Finally, we present a proof-of-concept measurement, laying the groundwork for future deployment in more application-like environments.

## Theoretical conceptualization of the measurement principle

### LiDAR equations for narrow wavelength tuning

Consider a 1D medium that is probed with a collimated light source that emits light at wavenumber $\nu$, and a detector of given aperture at *r* = 0. The light source and the detector are coplanar and coaxial. Several scatterers, i.e. particles, semi-reflecting windows, and/or reflecting surfaces are distributed along the *r*-coordinate. Starting with the elastic backscattering LiDAR equation^21^, the power *P* of the signal incident upon the detector is the product of four terms:

1 $$P\left(r,\nu \right)=C\cdot G\left(r\right)\cdot {h}_{{\text{B}}}\left(r\right)\cdot T(r,\nu )$$

The parameter $C$ characterizes the performance of the employed instrumentation and depends on the emitted laser power, the detector area $A$, the duration of the laser pulse, and the detector efficiency. The geometric parameter $G(r)$ accounts for the detector solid angle as viewed from the scattering location, $r$. ${h}_{{\text{B}}}(r)$ quantifies the backscattering, while $T(r,\nu )$ covers transmission losses along the LOS, and is the principal quantity-of-interest.

The detected signal is a superposition of light scattered at various positions along the LOS. The information on the distance $r$ at which a signal component was scattered is encoded in the delay between emission and detection. Since a two-way transmission path must be considered, light emitted at $t$ reaches the detector at ${t}_{\text{d}}=t+2r/c$. As the measurement method considered in the present article is built on time-of-flight (ToF) LiDAR, it is practical to rewrite Eq. (1) in the time domain

2 $$I\left({t}_{{\text{d}}},\nu \right)={I}_{0}\left(t,\nu \right)\cdot \frac{A}{{r}^{2}}\cdot {h}_{{\text{B}}}\left(r\right)\cdot T(r,\nu )={I}_{0}\left({t}_{d}-\frac{2r}{c},\nu \right)\cdot \frac{A}{{r}^{2}}\cdot {h}_{{\text{B}}}\left(r\right)\cdot T(r,\nu )$$

The objective of the analysis is to deconvolve Eq. (2) to recover the information contained in $T(r,\nu )$. Since the received signal is a superposition of the contribution of all scatterers, Eq. (2) can be understood as an integral equation which is integrating “backwards” in time and forwards in space:

3 $$I\left({t}_{{\text{d}}},\nu \right)={\int }_{0}^{{t}_{{\text{d}}}\cdot c/2}{\text{d}I}_{0}\left({t}_{{\text{d}}}-\frac{2r}{c},\nu \right)\cdot G(r)\cdot {h}_{{\text{B}}}\left(r\right)\cdot T(r,\nu )\text{d}r$$

Equation (3) may be recast as a Volterra integral equation of the first kind (IFK). Let $r\, = \,\left( {t_{{\text{d}}} \, - \,t} \right) \cdot c,t\, = \,t_{{\text{d}}} \, - \,r/c,\text{d}r\, = \, - \,c\text{d}t$, so

4 $$I\left({t}_{{\text{d}}},\nu \right)=-{\int }_{{t}_{\text{d}}}^{{t}_{\text{d}}/2}\text{d}{I}_{0}[\left({2t-t}_{\text{d}},\nu \right)G\left[\left({t}_{{\text{d}}}-t\right)c\right]\cdot {h}_{{\text{B}}}\left[\left({t}_{\text{d}}-t\right)c\right]\cdot T\left[\left({t}_{\text{d}}-t\right)c,\nu \right]\text{d}t=c{\int }_{{t}_{\text{d}}/2}^{{t}_{\text{d}}}a\left(t,{t}_{\text{d}}\right){f}_{\nu }\left(t\right)\text{d}t$$

where the unknown variable *f*_*ν*_*(t)* contains *G, h*_B_, and *T*. The kernel function, *a*(*t*,*t*_d_), is the laser waveform shifted through time. At time ${t}_{{\text{d}}}$, the detector is only sensitive to elements that fulfil the condition ${r}_{k}\le \left(c\cdot {t}_{\text{d}}\right)/2$. This horizon may also be characterized by a position-specific time ${t}_{k}$, i.e., the time required for the light to travel from the transceiver to the element at *r*_*k*_ and back to the receiver. If the difference ${t}_{\text{d}}-{t}_{k}$ is negative, the detector has yet to receive a signal from the $k$ th element. By discretizing the region-of-interest into small but finite segments of thickness $\Delta r$ and corresponding time intervals $\Delta t$, Eq. (4) may be approximated as

5 $${I}_{\nu }\left({t}_{\text{d}}\right)={\sum }_{k=1}^{{t}_{\text{d}}/\Delta {t}_{k}}{I}_{\nu ,k}={\sum }_{k=1}^{{t}_{\text{d}}/\Delta {t}_{k}}{I}_{0,\nu }\left({t}_{\text{d}}-{t}_{k}\right)\cdot {G}_{k}\cdot {T}_{\nu ,k}\cdot {h}_{{\text{B}},k}={\sum }_{k=1}^{{t}_{\text{d}}/\Delta {t}_{k}}{I}_{0,\nu }\left({t}_{\text{d}}-{t}_{k}\right)\cdot {f}_{\nu ,k}$$

The summation argument in Eq. (5) can be, analogue to Eq. (4), structurally divided into two factors: a discretised kernel function $a\left({t}_{\text{d}},{t}_{k}\right)={I}_{0,\nu }\left({t}_{\text{d}}-{t}_{k}\right)$ which is the waveform of the emitted laser pulse shifted through time, and the unknown variable ${f}_{\nu ,k}={f}_{\nu }\left({t}_{k}\right)={G}_{k}\cdot {T}_{\nu ,k}\cdot {h}_{{\text{B}},k}$ which contains the sought-after information on the absorption properties of the gases between the $k$th element and the transceiver. The geometric factor ${G}_{k}$, which is evaluated at each corresponding ${r}_{k}$, depends on the detector sampling rate and is known beforehand. The location ${r}_{k}=c\cdot {t}_{{\text{d}},k}/2$ is then the furthest point that can contribute to the signal at a given detection time *t*_d,*k*_.

Equation (5) may be transformed into a matrix equation. Let ${t}_{{\text{d}},{\text{max}}}$ be the longest detection time in the experiment and split into $K={t}_{{\text{d}},{\text{max}}}/\Delta t$ elements. The measurement vector ${I}_{\nu }={\left[{I}_{\nu }\left({t}_{\text{d}}=1\cdot \Delta t\right),{I}_{\nu }\left({t}_{\text{d}}=2\cdot \Delta t\right),\dots {,I}_{\nu }\left({t}_{\text{d}}=K\cdot \Delta t\right)\right]}^{T}$ consists of the detected intensities at each time step. Each element ${I}_{\nu ,j}={I}_{\nu }\left({t}_{\text{d},j}=j\cdot \Delta t\right)$ is given by:

6 $${I}_{\nu ,j}={\sum }_{k=1}^{k=j}{I}_{0,\nu }\left(j\Delta t-k\Delta t\right)\cdot {G}_{k}\cdot {T}_{\nu ,k}\cdot {h}_{B,k}={\sum }_{k=1}^{k=j}{A}_{\nu ,j,k}\cdot {f}_{\nu ,k}$$

Writing Eq. (6) for each detection time forms a matrix equation, **A**_ν_**f**_ν_ = **I**_ν_. Solving the system of equations amounts to deconvolving Eq. (6) for the field variable **f**_ν_. The vector **f**_ν_ $\in {R}^{Kx1}$ contains the information about the amount of the reflected light as well as the position of the reflectors. Note that this is a linear matrix equation. The overall aim is to deconvolve for the continuous field variable *f*_ν_(*r*) in a discretized manner. Since deconvolution of a Volterra IFK is ill-posed, inversion of this matrix equation requires regularization, as described more thoroughly in the Appendix.

### Reflection parameter

The scattering elements used in this experiment may be categorized into two classes: spatially discrete reflecting interfaces like the surfaces of optical elements and metal surfaces, and continuously distributed scatterers like particles and droplets entrained in a flow. In this case, the reflection parameter ${h}_{{\text{B}}}$ depends on their local properties as well as on the distance between the reflector and the transceiver through the changing detection solid angle $\Delta \Omega$. For particles, the reflection parameter ${h}_{\text{B}}$ can be modeled using the scattering parameter ${\sigma }_{{\text{s}}}$ and the scattering phase function ${\Phi }_{{\text{scat}}}$. For particles that are large compared to the wavelength of the light, the reflection parameter can be calculated as^22^

7 $${h}_{{\text{B}}}\left(r\right)\approx \frac{{\sigma }_{\text{s}}\left(r\right){\Phi }_{\text{scat,B}}}{4\pi }\Delta \Omega \left(r\right)={C}_{{\text{B}}}\left(r\right)\mathrm{\Delta \Omega }(r)$$

with the backscattered amount of the phase function ${\Phi }_{\text{scat,B}}$ incorporated into a space-dependent parameter, ${C}_{{\text{B}}}(r)$. The transmission $T$ quantifies the attenuation of the light due to absorption in the gas phase (absorption coefficient ${\kappa }_{\nu ,{\text{g}}}$), by the particulate matter (effective absorption coefficient ${\kappa }_{{\text{p}}}$) and scattering by particles (scattering coefficient ${\sigma }_{{\text{s}}}$). Absorption and scattering by droplets or particles are broadband in nature and can be modelled as spectrally independent over the narrow wavelength range spanned by the tuned diode laser. Consequently, only the gas phase absorption varies with the wavelength. The transmittance experienced by the light backscattered at a specific location *r* is given by the Beer-Lambert law^23^:

8 $$T\left(r,\nu \right)={\tau }_{\nu }^{2}={\text{exp}}\left(-2{\int }_{0}^{r}{\kappa }_{{\text{g}}}\left(\widetilde{r},\nu \right)+{\kappa }_{{\text{p}}}\left(\widetilde{r}\right)+{\sigma }_{{\text{s}}}\left(\widetilde{r}\right) \text{d}\widetilde{r}\right)$$

## Merging TDLAS and ToF-LiDAR

A unique aspect of this technique compared to other approaches described in the literature is the use of time-modulated tunable diode lasers to interrogate the spectroscopic properties of the gas, which is connected to the local thermodynamic state of the gas, and to also distinguish absorption by the gas phase from the broadband properties of the reflectors.

**Figure 1:**
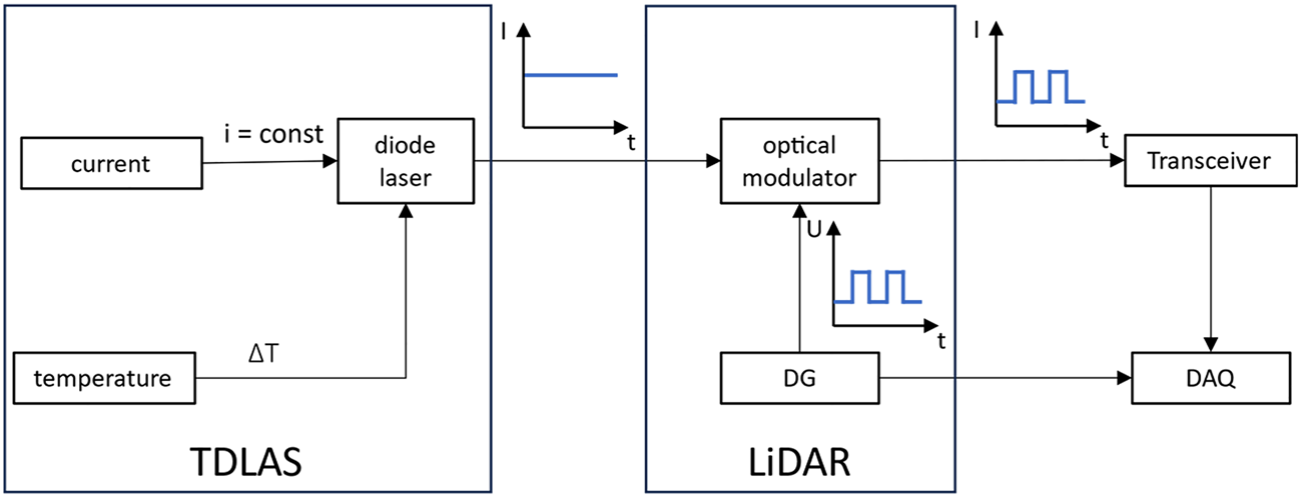
Experimental Setup for the combination of TDLAS and LiDAR: a diode laser operated in cw-mode is tuned in wavelength by temperature variation while the injection current is held constant. The light is led through an optical modulator to chop the laser cw-light into sharp pulses. The pulsed signal is sent through the region of interest. The backscattered signal is detected by a detection system of high bandwidth. Legend: DG: delay generator; DAQ: data acquisition system.

ToF LiDAR requires that the interrogation beam is modulated into waveforms having rise and fall times on the order of 100 ps to achieve a sub-centimeter spatial resolution, which cannot be done through current or temperature tuning methods since these operate at considerably longer timescales. Instead, the tunable diode laser is operated in cw-mode and paired with a fast-switching optical modulator (e.g., an electro^20,24^ or an acousto-optical modulator^17^) to generates the pulses, as shown in Fig. 1. The laser diode is tuned between the pulses by varying the injection current or temperature. Current tuning enables periodic scanning at kHz rates, but has the disadvantage that the intensity varies strongly along the wavenumber axis^6^. Since the backscattered signal is expected to be relatively weak, the laser is tuned by varying the diode temperature, ensuring that the emitted light intensity is maximized for each wavenumber. The rise time of the modulator determines the achievable spatial resolution.

After deconvolution, a spectrum is obtained for each point with a reflecting or scattering source in the region of interest. This spectrum contains the path-integrated information about the gas phase properties between the transceiver and the respective reflector. The local information is then extracted by an onion-peeling like approach: the spectra are fitted sequentially, starting with the spectrum associated to the shortest LOS. The difference between neighboring spectra corresponds to the local spectra that contain the sought-after information on the local gas properties. Finally, the local concentration, temperature, and potentially pressure of the gas may be inferred by a spectroscopic fit using the HITRAN database^25^. A more detailed explanation on TDLAS is given in^6,7,26^.

## Results

To establish the theoretical viability of the presented method, we consider two scenarios. The first scenario consists of two gas volumes of different composition separated by a window and terminated by a reflecting surface, which is analysed both numerically and experimentally. The second scenario is a particle-laden gas of spatially varying temperature, which is examined through simulation.

### Scenario 1: two gas volumes separated by a window

Consider the case comparable to the experimental setup shown in Fig. 4 consisting of two volumes separated by a window and terminated by a reflecting surface. This case is inspired by a pressurized combustion test apparatus consisting of an inner container that contains the combustion process to be investigated, enveloped within an outer pressure vessel which contains cooling air of unknown thermodynamic state^27,28^. The task is to measure the humidity of the inner container such that the measurement result is not disturbed by the cooling flow.

The two gas regions have lengths of *L*_1_ = 0.4 m and *L*_2_ = 0.1 m and are separated by a window of thickness *L*_w_ = 0.005 m. Both gas volumes are at ambient conditions (20 °C, 1 bar) but differ in humidity: the mole fraction of H_2_O in Gas 1 ranges from 0.4 to 1.6%, while that of Gas 2 is 1.3% for all cases. These scenarios are summarized in Table 1. For this purpose, we wish to find the transmittance spectra across the two gas volumes, ${\tau }_{\nu ,1}$ and ${\tau }_{\nu ,2}$, in the spectral domain between 7180 and 7186 cm^−1^ (1391.6 to 1392.8 nm), corresponding to several prominent absorption lines. The window has a reflectance ρ = 0.06 at each interface. The optical properties of the window and the reflecting surface are approximately constant over the considered wavenumber domain. In the absence of particles, scattering is negligible, i.e. ${\sigma }_{{\text{s}}}=0$.

**Table 1:** Simulation results: set mole fraction versus reconstructed mole fraction.

Simulation no.			I	II	III	IV	V
Box (Gas 1)	*q*_H2O,1,set_	[%_mol_]	$0$	$0.400$	$0.800$	$1.20$	$1.60$
	*q*_H2O,1,fit_	[%_mol_]	$6.86\cdot {10}^{-3}$	$0.405$	$0.804$	$1.20$	$1.60$
	abs. error	[%_mol_]	$6.86\cdot {10}^{-3}$	$5.33\cdot {10}^{-3}$	$3.80\cdot {10}^{-3}$	$2.26\cdot {10}^{-3}$	$7.32\cdot {10}^{-4}$
Cell (Gas 2)	*q*_H2O,2,set_	[%_mol_]	$1.30$	$1.30$	$1.30$	$1.30$	$1.30$
	*q*_H2O,2,fit_	[%_mol_]	$1.28$	$1.29$	$1.30$	$1.31$	$1.32$
	abs error	[%_mol_]	$2.30\cdot {10}^{-2}$	$1.32\cdot {10}^{-2}$	$3.39\cdot {10}^{-3}$	$6.43\cdot {10}^{-3}$	$1.63\cdot {10}^{-2}$

The intensity propagation through the gas is governed by the time-dependent radiative transfer equation (RTE)^22,29,30^, which is solved using a finite volume code as described in the appendix. Gaussian noise with a noise level of 50 dB is added to both the simulation data and the laser form function. This SNR would be typical of what would be achieved by averaging 35,000 individual waveforms using the experimental apparatus described in the next section. Simulations of the measured intensity response are performed for 301 equally-spaced wavenumbers between 7180.62 and 7183.46 cm^−1^ (1392.1–1392.6 nm). The spectra from both reflectors are then reconstructed from the simulated intensity profiles. Finally, the gas properties are determined by a spectroscopic fit.

**Figure 2:**
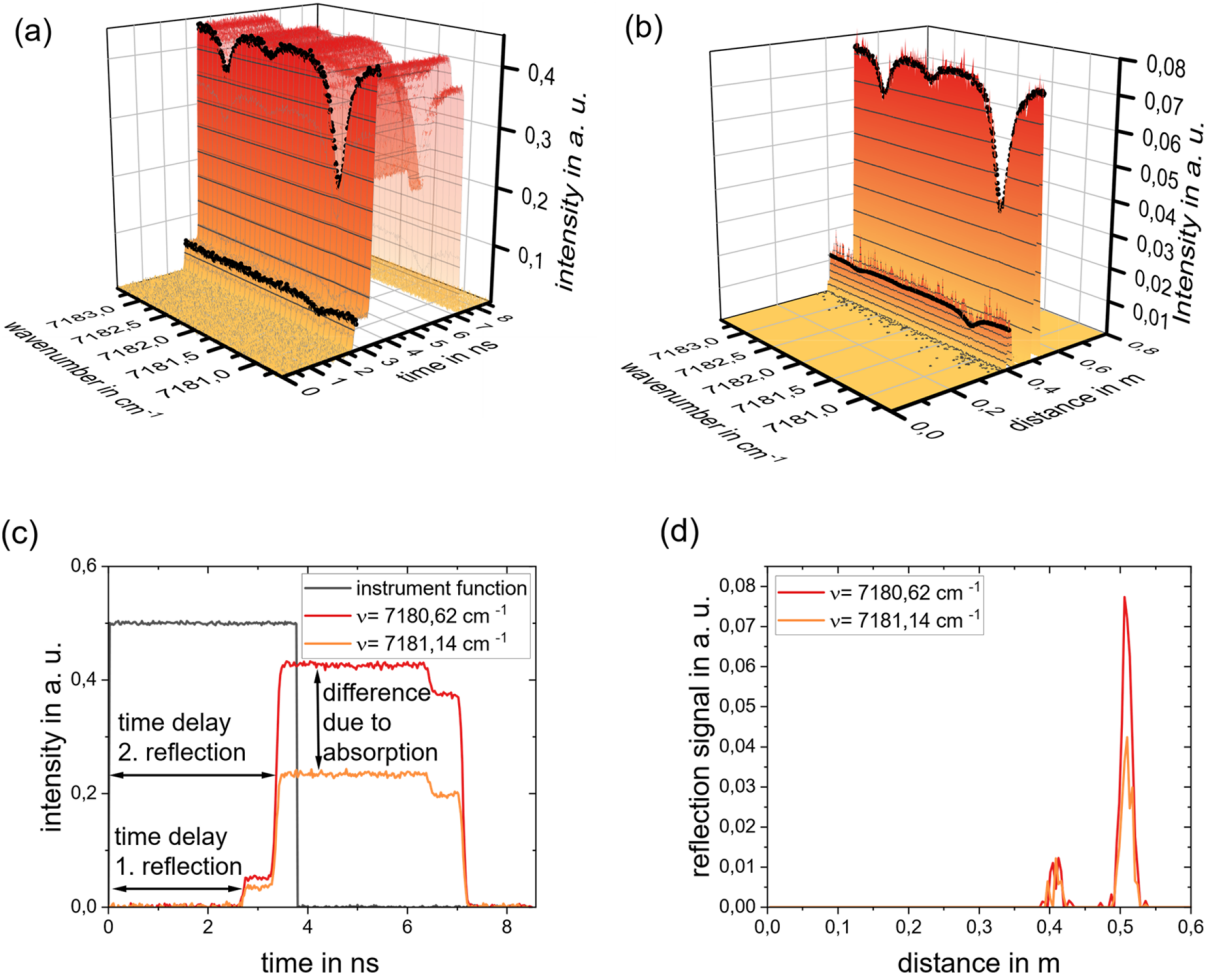
Result of the finite volume simulation for discrete reflectors for configuration II. (**a**) Simulated detected signal plotted over the wavenumber and space axis: box-shaped signal because of the convolution of the reflection with the laser form function, black traces are included to highlight the spectra in the signal; (**b**) deconvoluted detected signal: clear separation of the signals in space; (**c**) Simulated intensity traces. Black: instrument function, red: detected signal without absorption (off-resonant wavelength), orange: detected signal with absorption (resonant wavelength). (**d**) Result of the deconvolution of the detected intensity traces with the instrument function.

Figure 2a shows the recorded intensity over each simulated wavelength. The box-shaped appearance originates from the temporal pulse function of the emitted laser beam. The two separated reflections are highlighted with black traces. The discrete distribution of the reflectors becomes clearer after the deconvolution according to Eq. (5), see Fig. 2b. The time axis is transformed to the space axis using $\text{d}r={c}_{0}\cdot \text{d}t/2$. After the deconvolution, the analysis of the absorbance spectra can be performed.

Figure 2c compares simulations made at two exemplary wavenumbers, one resonant and the other off-resonant. The optical pathlength can be inferred from the time difference between the detection of the reflections and the emission of the laser pulse form. This becomes clear by examining the result of the deconvolution of the signals with the laser form function, Fig. 2d. The position of the peaks indicates the location of the corresponding reflector, while the peak magnitude depends on the reflection coefficient of the reflector and the optical depth of the path between reflector and transceiver. In principle, as the reflectance of a surface appears on a distinct location in space, the reconstructed reflection should appear as a delta function. However, due to the finite acceptance angle of the receiver, or if the windows are not exactly normal, the reflected light will travel a distribution of distances back to the detector, resulting in a narrow temporal distribution of detection. Figure 2d shows that reflection from the mirror ($r\approx 0.5 {\text{ m}}$) is very distinct, while the reflection from the window ($r\approx 0.4 {\text{ m}}$) is smaller and broader due to higher numerical diffusion at lower signal strength and the stronger influence of noise. As the reflections are clearly separated from each other, the influence of small temporal deviations in the signal and therefore in the solution of the deconvolution given in the appendix, can be minimized by summing the solution vector over a small region around the peak location that were found in the previous analysis step.

**Figure 3:**
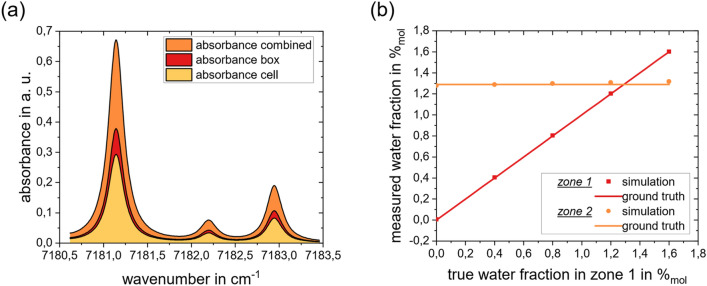
Results for Simulation: (**a**) Case II: successively calculated absorbance spectra for the same data. Note that the total absorbance corresponds to the superposition of the absorbances in the box and the cell, i.e., ; (**b**) Comparison of the inferred water mole fractions in the two regions with the respective ground truth used to generate the data.

The inferred absorbance spectra are shown in Fig. 3. Gas properties are then obtained by regressing modelled spectra derived using HITRAN^25^ to the inferred spectra. The absorbance of the gas in Zone 1 (reflection at *r* ≈ 0.4 m, low water concentration, but long absorbance path) is calculated first using the first back-reflected signal component; this value is then used to obtain the absorbance of Gas 2 (gold area) using backscatter at longer measurement times. The results for all five simulated conditions are summarized in Table 1.

Figure 3b visualizes the results of the inference for a total of five simulations conducted for the conditions listed in Table 1. It is evident that the varying water concentration is detected in Zone 1, while the inferred composition of the gas in Zone 2 remains constant and is not affected by the changes in Zone 1. The accuracy of the inferred water mole fraction for Zone 2 is lower than Zone 1, due to error amplification of the ill-posed system. Nonetheless, the gas compositions in each zone may be distinguished clearly.

**Figure 4:**
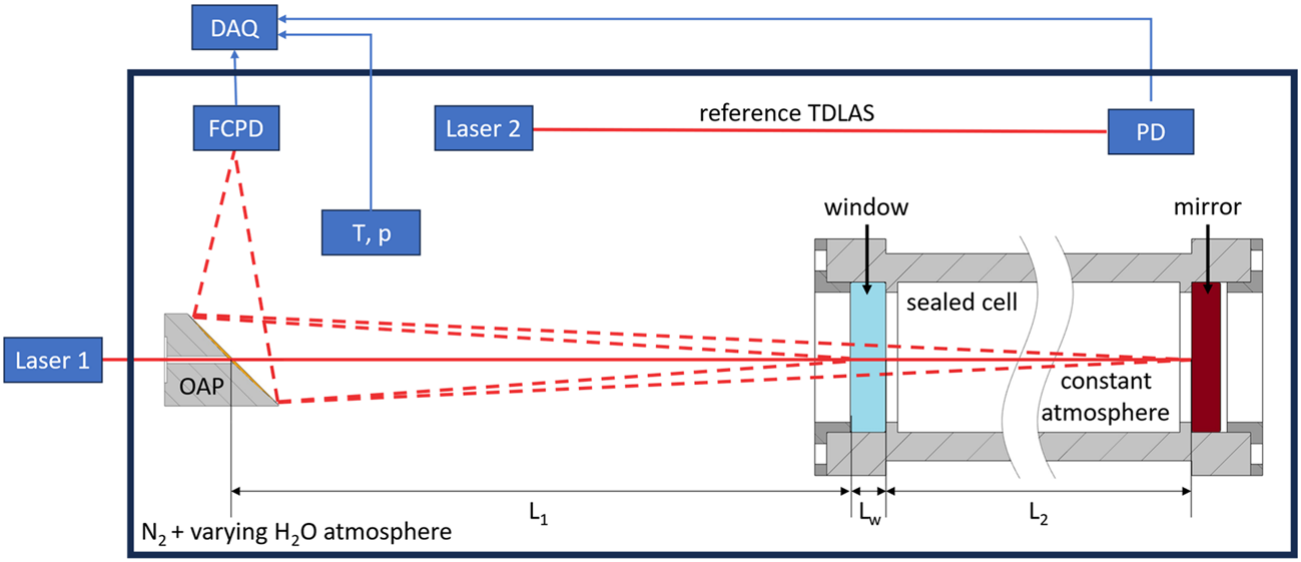
Experimental schematic: Temperature tuned Laser 1 is lead through an off-axis parabolic mirror (OAP) in the ROI. Reflections from the window and mirror at the sealed cell are collected by a high bandwidth fiber coupled photodiode (FCPD) and analyzed in the DAQ-system. For reference humidity measurements a second TDLAS system with current tuning is build up inside the varying atmosphere. Additional temperature and pressure sensors are placed inside the ROI. Not that the atmosphere in the sealed cell is held constant while the outer atmosphere is changed between several measurements. The relevant pathlengths are the absorbance pathlength in the other box L_1_ and inside the cell L_2_ completed by the window thickness L_w_.

Figure 4 shows the experimental realization of this scenario, consisting of a sealed gas cell enveloped within a larger sealed volume that also contains the optical elements. On the side facing the instrumentation, the cell is optically accessible through a wedged sapphire window ($L_{w} = 5\;{\text{mm}}$), while the optical path is terminated by a metal reflector. The distance between the emitter and the outer surface of the window is $L_{1} = 38.54\;{\text{cm}} \pm 0.14\;{\text{cm}}$, while the inner surface of the window and the reflector define the optical path within the cell, $L_{2} = 10,03\;{\text{cm}} \pm 0.11\;{\text{cm}}$. The objective of the experiment is to infer the water content of both gas volumes. The composition of the inner cell is constant over all measurements, with a water mole fraction of $1.31{\text{\%}}_{\text{mol}}$. The moisture content of the enveloping volume is adjusted using a flow of dry nitrogen. The atmosphere within the box is monitored using an independent single-pass TDLAS system, along with pressure and temperature sensors (Tinkerforge bricklets). The considered operating points are summarized in Table 2.

**Table 2:** Overview of the experimental parameters.

Measurement no.		I	II	III	IV	V
*q*_H2O,1_	[%_mol_]	0.46	0.44	0.05	1.45	1.48
*q*_H2O,2_	[%_mol_]	1.31	1.31	1.31	1.31	1.31

**Figure 5:**
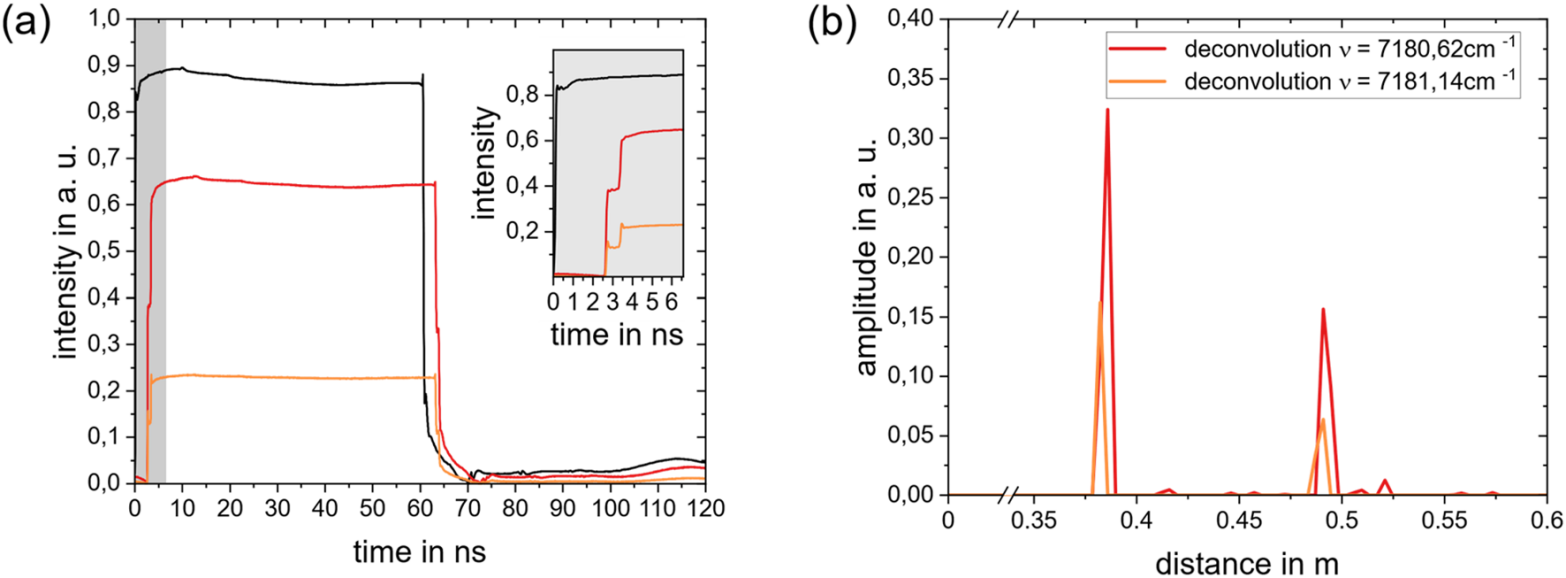
Measurement results. (**a**) Measured intensity traces. Black: instrument function showing the shape of the emitted pulses, red: detected signal at an off-resonant wavelength, orange: attenuated signal acquired at a resonant wavelength. (**b**) Result of the deconvolution of the intensity traces with the instrument function.

The light source is a discrete mode diode laser (Eblana Photonics) operated in cw-mode and scanned between 7180.62 and 7183.46 cm^−1^ by changing the diode temperature. The laser beam is time-modulated using a fiber-coupled electro-optic modulator (Thorlabs LNA6213) controlled by a delay generator (Stanford Research Systems DG645 with SRD1). To provide sufficient power to drive the EOM, the signal is amplified using an RF amplifier (Mini-Circuits ZX60-14012L-S+). Figure 5a shows an example intensity profile generated with the described system (black solid line) with a rise-time of $t_{20 - 80} = 67.5\;{\text{ps}}$ and a fall-time of $t_{80 - 20} = 239.7\;{\text{ps}}$. The pulses have a length of 62 ns and are generated at a frequency of 6 MHz.

The light pulses are directed to the sensor head using glass fibers. Ten percent of the intensity is guided onto a photo diode via a fiber-coupled beam splitter for a reference measurement. The light is then decoupled using a collimator centred within a perforated off-axis parabolic (OAP) mirror. The collimator images the light onto the gas cell window, while the OAP mirror focuses the reflected light onto a fiber, through which it is guided onto a high-speed photo diode (Newport Newfocus 1544-B-50) attached to an oscilloscope (LeCroy WaveMaster 820Zi-A) with which the intensity trace is sampled at a rate of $40\;{\text{GHz}}$. The measurement duration is limited by the high-speed memory of the oscilloscope, which can store $256 \cdot 10^{6}$ datapoints at a time. This limits the time to a total of $6.4\;{\text{ms}}$, before the data needs to be transferred via an ethernet cable to the computer where it is preprocessed.

Exemplary averaged intensity traces are plotted Fig. 5a, which resemble the simulation results. The temporal delay between the emission of the pulse and the detection of the first reflection due to the finite speed of light is clearly visible. The pronounced steps in the rising edge of the detected trace corresponds to the temporal delay between the incidence of the reflections from the window and the metal surface on the detector. While the intensity trace displayed in red was acquired at an off-resonant wavelength, the orange one corresponds to a resonant wavelength and thus features considerable absorption, reducing the intensity of the reflections.

In Fig. 5b, the deconvolved $h_{{\text{B}}}$ values indicating reflectors at positions $r \approx 38\;{\text{cm}}$ and $r \approx 49\;{\text{cm}}$, which correspond to the window and the reflector, respectively. The height of the peaks indicates that more light is reflected from the window onto the detector than from the metal surface. Due to the attenuation by absorption, the peak of the resonant wavelength (orange) is lower than those at off-resonant wavelength (red).

**Figure 6:**
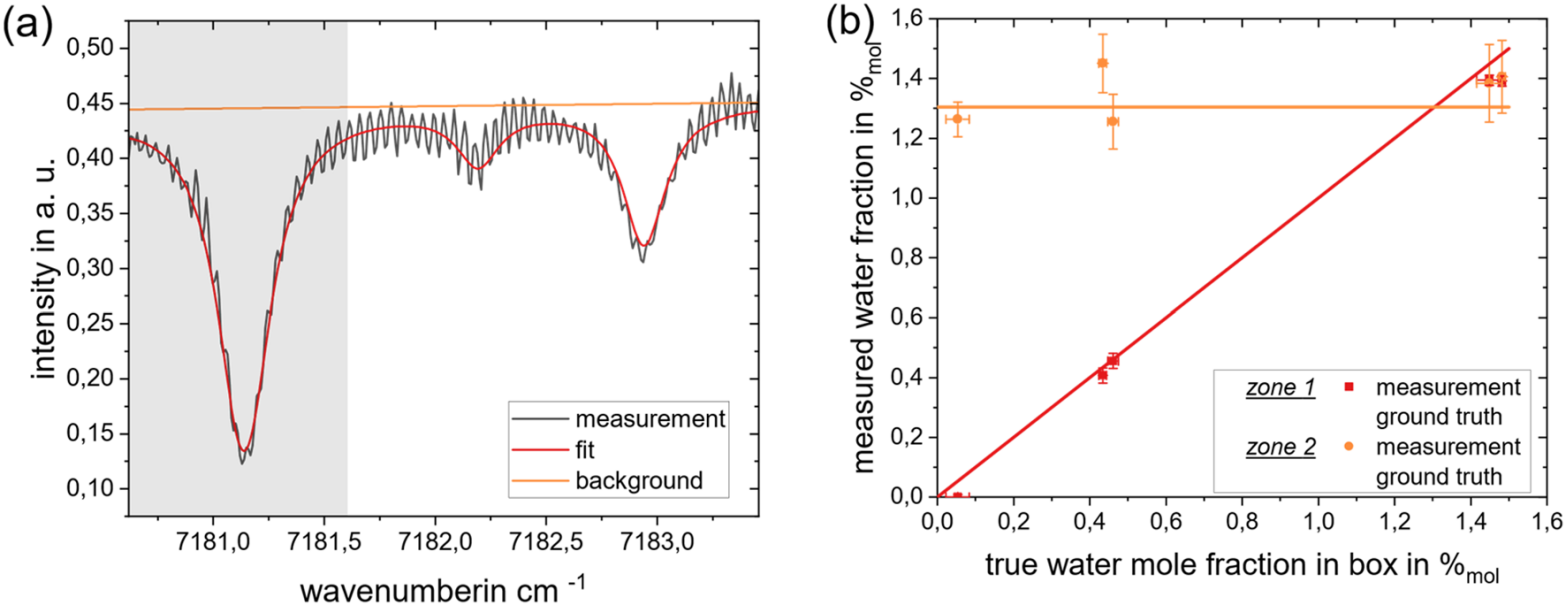
Measurement results: (**a**) Exemplary spectrum resulting from the measured reflections from a reflector (black solid line). In addition, the spectroscopic fit (red) and the background (orange) are plotted; (**b**) Mole fraction for the different regions (■: box, ●: cell).

If the peaks associated with a specific reflector are plotted for all recorded wavenumbers, an intensity spectrum like that shown in Fig. 6a (black solid line) is obtained. Water absorption lines are immediately recognizable, along with high-frequency fringing caused by the orthogonal alignment of the beam to the optical elements. The states of the gas within the cell and inside the enveloping chamber are quantified using the wavenumber range highlighted in gray. Since the intensity dependence is significantly lower in temperature tuning, the order of the background polynomial was set to 1 for this study using a reference measurement.

The result of the measurements at the operating conditions listed in Table 2 is plotted in Fig. 6b. Solid lines indicate the expected water concentrations in Zone 1 (red) and Zone 2 (orange). The red squares and orange circles denote the reconstructed water mole fraction in box and cell, respectively. Horizontal error bars quantify the variation of the water mole fraction in the box during the measurement time and include 90% of the values measured by the reference TDLAS. The vertical error bars indicate the uncertainty of the spectroscopic fit performed to infer the water mole fractions from the deconvolved intensity spectra following Emmert et al.^31^. Moisture measurements reconstructed in the outer enveloping volume are quantitatively robust; those within the gas cell follow the expected trend but show larger variation from the ground-truth. The measurement results for the box are more robust than those for the cell because the error amplification for the rear reflector is significantly larger. This, in turn, is due to the more ill-posed character of the evaluation. This relation of the data evaluation procedure is also observed in the above simulation study.

### Scenario II: continuous distribution of scatterers

We now consider the potential of the technique for analyzing continuously scattering gas volumes using a simulation study to generate spatially resolved gas phase absorption spectra. It is assumed that the process is steady, as is the case in industrial combustors for coal or biomass combustion.

**Figure 7:**
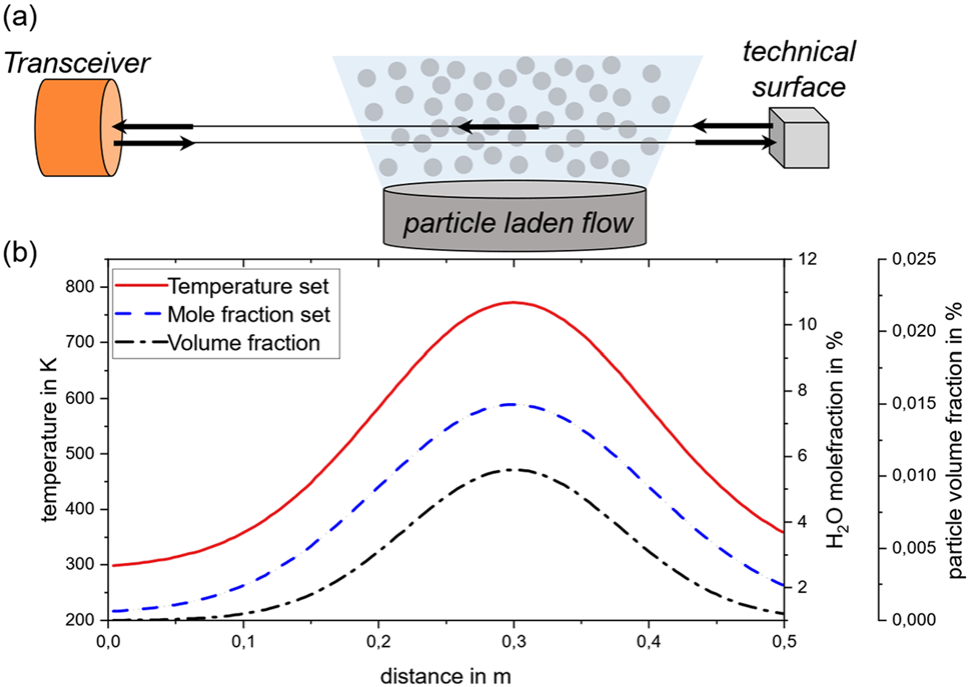
Setup for the FV simulation with a particle laden flow: (**a**) domain description (I_0_: incident intensity; I_B_: backscattered intensity; I_D_: detected intensity), (**b**) Temperature, H_2_O mole fraction and number of particles per volume unit distribution over the domain length *r*.

Consider the case shown in Fig. 7 (a). The aerosol emerges from a 0.2 m diameter pipe placed at a distance 0.2 m away from the transceiver. The considered domain has a width of 0.5 m. The flow has a peak temperature of approximately 500 °C and a maximum water mole fraction of 7.6%. The particle cloud is defined with a volume fraction of up to 0.01% for particle diameters of ${d}_{\text{particle}}=100$ µm. The flow emerging from the pipe gradually entrains the surrounding atmosphere, resulting in the distributed properties shown in Fig. 7b.

Because the particles are large compared to the laser wavelength, they may be modeled as diffusely-reflecting spheres. This example considers wood as a biomass with an absorptance ${\alpha }_{\text{p}}=0.75$. A more detailed explanation of the used particle reflection model is given in the appendix. The in-scattering or the local reflection coefficient ${h}_{\text{B}}\left(r\right)$ multiplied by the local incident radiation $I\left(r\right)$ can by calculated by Eq. (7). The measurement path is terminated by a diffuse surface having a reflectance of 5%.

**Figure 8:**
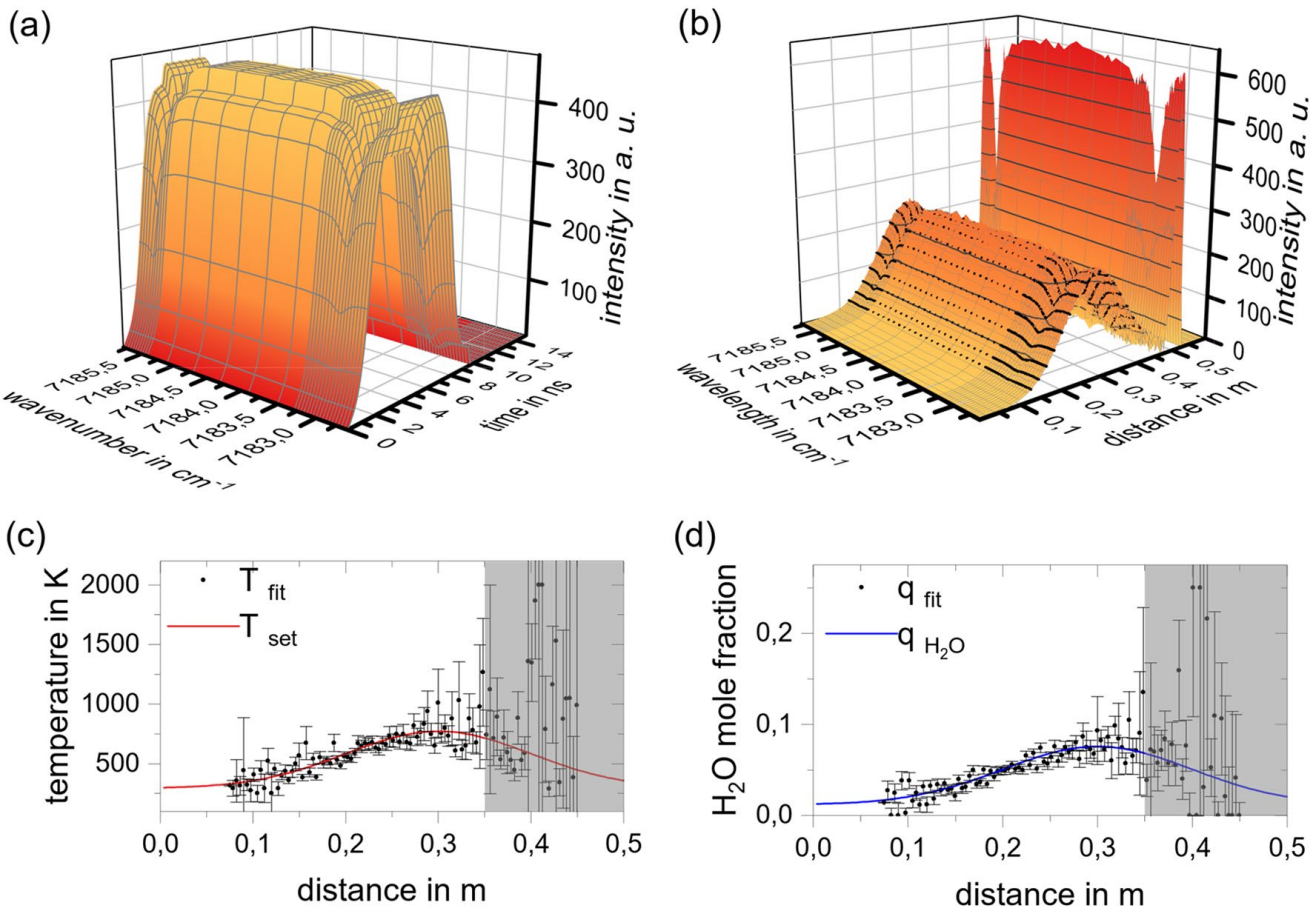
Result of the finite volume simulation for a particle laden flow. (**a**) Simulated detected signal plotted over the wavenumber and space axis: box-shaped signal because of the convolution of the reflection with the laser form function; (**b**) deconvoluted detected signal: reflection at the particle surfaces distributed over the special domain, black traces highlight reflection at selected locations to show the effect of the gas absorbance; (**c**) spectral fit solved for the local gas temperature; (**d**) spectral fit solved for the water vapor mole fraction.

Figure 8 shows the simulated signal for this scenario. Due to the convolution of the reflection with the rectangular laser pulse (see black curve Fig. 2c), the signal in Fig. 8b appears as an accumulation of the individual reflections. The simulated detected signal is contaminated with Gaussian noise to represent the experimental noise. For this study an SNR with an amplitude of 100 or 20 dB is used. Since the process is assumed to be steady, multiple waveforms may be averaged to improve the SNR, even if the signal amplitude is comparable low. As we aim to infer mean absorbance spectra by averaging over multiple pulses, higher SNR can be traded for longer acquisition times. This value is conservatively estimated from the performance of commercially available equipment that would be suitable for measurements using the described approach.

The particles positioned closer to the transceiver generate a stronger signal due to the larger solid angle subtended by the detector when viewed from these particles. To eliminate this effect the signal contribution is divided by the solid angle at a given location. The temporal distribution of the signal contains information concerning the spatial distribution of the particles, while information about the local gas temperature and composition is contained in the absorption spectrum. The deconvolution is performed with a least square algorithm, see Appendix. The computed particle reflection function, ﻿*h*_B_(*r*), correlates with spatial distribution of the volume fraction (see Fig. 7). The influence of the local gas temperature and gas mole fraction is included in the wavelength-dependent absorbance features. The black traces in Fig. 7b highlight selected intensity traces over the wavenumber axis to emphasize the absorbance of the gas species.

The reflected signal starts to accumulate at a transceiver distance of approximately $r = 0.1\;{\text{m}}$. Starting at that point, the transmission spectra over the wavenumber is evaluated with a spectral fit for the temperature and the water vapor mole fraction until the signal vanishes again at a distance of $r \approx 0.45\;{\text{m}}$. There are several absorption lines within the wavenumber axis for water vapor. The result of the spatial distributed spectral fit regarding the local temperature and mole fraction of water vapor are included in Fig. 8c,d, demonstrating the viability of this approach, although the signal to noise ratio and error amplification play an important role. The fit shows higher uncertainties at the spatial start of the particle cloud and falls at the end of the cloud. The higher uncertainty at close locations has its origin in the small SNR. Since the measured signals are analyzed by an onion peeling-like approach, error propagation is evident in the reconstructed parameters. At more distant locations, higher uncertainties arise, as they are propagated from the previous fits. This is why fit uncertainties are significantly higher from *r* > 0.35 m, such that this region can no longer be evaluated (compare grayed area in Fig. 8c,d). Nevertheless, spatially resolved gas temperature and water mole fraction measurements for over 60% of the spatial distribution of the particle cloud can be performed. At $r < 0.1\;{\text{m}}$, the particle density is not sufficient for meaningful reconstructions, while for $r \ge 0.35\;{\text{m}}$ the limiting factor is induced by the error amplification.

## Conclusions

This article presents a novel measurement approach that merges tunable diode laser absorption spectroscopy with optical ranging to measure gas phase properties in particle-laden flows with high spatial resolution. Compared to established measurement techniques, the proposed method has the major advantage that it only requires minimal optical access to the investigated process and uses inexpensive tunable diode laser sources to resolve the spectra. The viability of this technique is assessed in two scenarios. The first application case focused on inferring the water mole fractions in two volumes partitioned by a glass wall, which was explored through simulation and physical experimental analysis. The second application case treats measurements in particle laden flows, where instead of two sections, the absorbance spectra and thermo-chemical properties are inferred. A numerical study revealed that practical measurements in particle-laden flows are possible provided that the signal-to-noise ratio of the detected signal is sufficiently large. However, various influences on SNR like beam-steering, stochastic scattering by moving particles, and realistic losses at process windows need to be evaluated in a future study.

The presented results underscore the potential of merging TDLAS with optical ranging to infer local gas phase properties in particle-laden flows and lay the theoretical foundation for advanced measurements. Future work will focus on conducting an experimental proof-of-concept for the particle-laden flow and the optimization of the experimental setup to perform measurements in technically relevant scenarios.

## Data Availability

The datasets used and analysed during the current study available from the corresponding author on reasonable request.

## Data evaluation

The position of reflectors in the beam path can be determined by measuring the time delay between the emission of a light pulse and the detection of reflections. Starting with the matrix formulation in Eq. (6), the overall aim is to deconvolve for the field variable *f*_*ν*_(*r*). Note that this is a linear matrix equation and that a reflection can only be greater than or equal to zero. To get the position of the reflections a deconvolution has to be performed, for which regularization is needed due to the ill-posedness of this process. For cases with discrete reflectors, the deconvolution of the field variable ***f***_*ν*_ is performed with a nonnegativity constraint^32^:

9 $$\underset{f\in {R}^{n}}{min}\left[{\left|\left|{{\varvec{A}}}_{\nu }{{\varvec{f}}}_{\nu }-{{\varvec{I}}}_{\nu ,\text{D}}\right|\right|}_{2}^{2}\right]s.t.{f}_{\nu ,i}\ge 0\,{\text{for}}\,i\in \{1,\dots ,n\}$$

The positions of the reflectors are aligned with the *p* largest elements of the reflection vector *h*_B_(*r*), while *p* is chosen according to the expected number of reflectors. In addition, the knowledge that the reflectors are spatially separated can be used to increase the robustness of the evaluation by binning the signal in their vicinity.

In case of a continuous reflecting region of interest, such as in particle laden flows, a smooth solution is preferred, reflecting the prior knowledge of continuous distribution of reflectors. To achieve this the penalty term ${\gamma }_{s}{\left|\left|{{\varvec{L}}}_{2}{{\varvec{f}}}_{\nu }\right|\right|}_{2}^{2}$ with a regularisation parameter ${\gamma }_{s}$ and a second order finite difference matrix **L**_**2**_ is implemented as follows^33,34^:

10 $$\underset{f\in {R}^{n}}{min}\left[{\left|\left|{A}_{\nu }{f}_{\nu }-{I}_{\nu ,\text{D}}\right|\right|}_{2}^{2}+{\gamma }_{\text{s}}{\left|\left|{L}_{2}{f}_{\nu }\right|\right|}_{2}^{2}\right]s.t.{f}_{\nu ,i}\ge 0\,{\text{for}}\,i\in \{1,\dots ,n\}$$

To get the most meaningful results out of the deconvolution the regularisation parameter must be chosen appropriately. In order to choose the right regularization parameter, an L-curve is calculated^35^, and the regularization corner is taken as the regularization parameter ${\gamma }_{\text{s}}=0.7$. The deconvolution is performed for each of the measurements at $w$ different wavenumbers.

## Simulation: finite volume method

The finite volume simulation is performed using the MATLAB toolbox from Eftekhari et al.^36^. Intensity propagation through the gas is governed by the time-dependent radiative transfer equation (RTE)^29,30^:

11 $$\frac{1}{c}\frac{\partial {I}^{lm}}{\partial t}+\frac{\partial {I}^{lm}}{\partial {r}^{l}}=-{\beta }_{\nu }^{l}{I}^{lm}+{S}^{lm}$$

where *r* is the discrete path-variable in each defined region, $m\in \{\mathrm{1,2}\}$ serves as an index for the angular dependence of the transmitted light and $l\in N$ is an index for the different regions. Since all light/surface interactions are taken to be specular, the intensity need only be solved in the forward (*m* = 1) and backward (*m* = 2) propagation directions. Variable ${\beta }_{\nu }$ symbolises the extinction coefficient and S is the source term. The source term has to be treated differently according to the simulated case. With only discrete scatterers along the beam path, *S* is used as a boundary condition term between the *l* regions. When a lot of scatterers are located inside the domain and form a continuous scattering cloud, as e. g. in solid fuel combustion or in general particle laden flows, *S* should be taking account to the in-scattering term. A discussion of the implementation of the source term *S* is given below, together with a discussion on the chosen temporal and special discretization. The results presented here have been obtained by simulating the RTE on the presented domain with time steps of $\Delta t=0.125 {\text{fs}}$ and cell sizes of $\Delta r=0.2 {\text{mm}}$ which results in a courant number ${\text{Co}}<0.02$.

## Simulation: source term S

To further discuss the source term *S,* we must discretise the domain. Since the domain is one-dimensional in space, we divide each region in several finite volumes with an equidistant length $\Delta r$. Depending on the size of each defined region, each region is discretized in $i\in \{1,\dots ,{p}_{n}\}$ control volumes. Further we discretize in the time domain in $j\in N$ intervals with a finite time difference $\Delta t$ between each timestep. The source term *S* is then coded as a boundary condition for each region and is defined for each time step as the following:

12 $$S\left( {t_{j} } \right) = \left\{ {\begin{array}{*{20}l} {R_{m} \cdot I\left( {r_{i - 1}^{m} ,t_{j - 1} } \right)} \hfill & {{\text{for }}i = p_{m} \forall m \in \left\{ {1, \ldots ,n} \right\},j \ge 2} \hfill \\ {1 - R_{m} \cdot I\left( {r_{{p_{m - 1} - 1}}^{m - 1} ,t_{j - 1} } \right)} \hfill & {{\text{for }}i = 1\forall m \in \left\{ {2, \ldots ,n} \right\},j \ge 2} \hfill \\ {\psi \left( {t_{j} } \right)} \hfill & {{\text{for }}i,\;m = 1,\forall j} \hfill \\ 0 \hfill & {{\text{else}}} \hfill \\ \end{array} } \right.$$

In the boundary definition $R_{m}$ is defined as the reflection coefficient of the technical surface (optical window, metal surface or mirror) if the $m$-th region. In principle is the reflection coefficient is dependent on the wavenumber $\nu$ of the incident laser light, since we only take respect to a small variation of the wavenumber, we consider the reflection coefficients as constant. The parameter $\psi \left(t\right)$ describes the trigger signal, which indicates the laser pulse at a certain wavenumber.

For the simulation of the scattering of particle laden flow the source term $S$ in Eq. (11) can no longer be handled as a boundary condition term between separately defined domains. The source term for this case must capture the in-scattering of the particles, as well for the origin direction of propagation of the irradiated light ($l=1$) and the reflected light ($l=2$). The particle reflections are modelled as diffusely reflecting spheres, as they are much greater than the laser wavelength. The absorption and scattering efficiencies equal to the absorptance and reflectance of the bulk interfaces, ${Q}_{\text{r}}={\alpha }_{\text{p}}$ and ${Q}_{\text{scat}}={\rho }_{\text{p}}=1-{\alpha }_{\text{p}}$. These efficiencies are connected to the bulk absorption and scattering properties of the particle phase by

13 $$\begin{gathered} \kappa_{\text{p}} \left( r \right) = \frac{{\pi d_{\text{p}}^{2} }}{4}N_{\text{p}} \left( r \right)Q_{\text{r}} = \frac{{\pi d_{\text{p}}^{2} }}{4}N_{\text{p}} \left( r \right)\alpha_{\text{p}} ; \hfill \\ \sigma_{\text{s,p}} \left( r \right) = \frac{{\pi d_{\text{p}}^{2} }}{4}N_{\text{p}} \left( r \right)Q_{\text{scat}} = \frac{{\pi d_{\text{p}}^{2} }}{4}N_{\text{p}} \left( r \right)\rho_{\text{p}} \hfill \\ \end{gathered}$$

Those coefficients, along with the gas absorption coefficient, are inserted into the extinction coefficient in Eq. (8). The simulated intensity is calculated using the phase function^22^:

14 $${\Phi }_{\text{scat}}\left(\theta \right)=\frac{8}{3\pi }\left[sin\left(\theta \right)-\theta cos\left(\theta \right)\right]$$

The only reflection coefficient that is computed differently is the reflection at the technical surface at the end of the domain. It is assumed, that the boundary will absorb most of the incident intensity. Only a small reflection of 5% will be diffusely reflected in the hemisphere in the direction of the transceiver. The transceiver will only detect the fraction of that according to the corresponding solid angle $\Delta {\Omega }_{end}$.
